# Exonic mutations of POU class 1 homeobox 1 are associated with milk pH in high-producing Holstein Friesian cows

**DOI:** 10.14202/vetworld.2024.2304-2310

**Published:** 2024-10-17

**Authors:** Muhammad Cahyadi, Ammar Ibnu Hasan, Djorodjatun Samodro Sakti, Nasta ‘Ainun Nissa, Ahmad Pramono, Suryo Firmanto, Rizwan Nur Friyatna, Slamet Diah Volkandari, Pita Sudrajad

**Affiliations:** 1 Department of Animal Science, Universitas Sebelas Maret, Surakarta, Jawa Tengah 57126, Indonesia; 2 PT Ultra Peternakan Bandung Selatan, Pangalengan, Kabupaten Bandung, Jawa Barat 40378, Indonesia; 3 Research Center for Food Technology and Processing, The Indonesian National Research and Innovation Agency, Gunung Kidul, Daerah Istimewa Yogyakarta 55861, Indonesia; 4 Research Center for Animal Husbandry, The Indonesian National Research and Innovation Agency, Bogor, Jawa Barat - 16911, Indonesia

**Keywords:** exonic mutation, Holstein Friesian, milk pH, physicochemical properties, *POU1F1*

## Abstract

**Background and Aim:**

Milk physicochemical properties play essential role in the milk processing industry, which are moderately to highly affected by genetic factors. This study aimed to evaluate the association between single nucleotide polymorphisms in POU class 1 homeobox 1 (*POU1F1*) and the physicochemical properties of milk in high-producing Holstein Friesian (HF) cows.

**Materials and Methods:**

A total of 149 high-producing dairy cows from PT Ultra Peternakan Bandung Selatan was included in this study. The physicochemical properties of milk, including density, freezing point, pH, lactose, solid non-fat, protein, and ash content, were determined. Moreover, three polymorphisms within the exon regions of *POU1F1* (c.195G>A, c.300G>T, and c.828G>A) were analyzed using polymerase chain reaction-restriction fragment length polymorphism. The association between these polymorphisms and the physicochemical properties of milk was determined using a mixed-effects model analysis, in which the lactation period was used as a covariate.

**Results:**

This study found that two polymorphisms, c.195G>A and c.828G>A, significantly affected the pH of fresh milk. Cows with both the GG genotypes c.195G>A and c.828G>A had lower milk pH values than those with the other genotypes. In addition, a non-significant effect of *POU1F1* was observed on the other physicochemical properties of milk.

**Conclusion:**

Two *POU1F1* polymorphisms determined the pH of fresh milk in the Indonesian HF population. These are potential marker candidates for milk pH that directly affect the development of dairy products in the milk processing industry.

## Introduction

Milk production and quality are economically important quantitative traits in the dairy industry. Most dairy industries depend on increased milk production in cattle breeds, such as Holstein Friesians (HFs) and milk quality consistency to meet the required standards for milk to be processed into various dairy products. Milk is a critical agricultural product with significant economic and nutritional value because it is well-known as a complete food comprising various nutritional compounds, including lactose, lipids, proteins, and microcomponents such as vitamins and minerals [1, 2]. In addition, the physical properties of milk, such as its density, pH, and freezing point, are important factors to be considered in the milk industry. These attributes play crucial roles in the technological and engineering aspects of milk processing, determination of milk microstructure, and understanding the complex chemical reactions occurring in milk [3].

Economically important traits such as yield and composition of milk produced by dairy cows are significantly affected by genetic factors. The effect of genetic factors on the rate of genetic change of quantitative traits is known as heritability, which is measured as the degree to which heredity affects a certain trait. Heritability quantifies the overall phenotypic variation attributable to genetic factors [4]. The heritability of milk yield and composition ranged from 0.25 to 0.50 (intermediate to high), indicating that these traits could be improved through proper genetic selection procedures. Selection procedures can improve the phenotypic value of a trait with changes in breeding values [5]. Therefore, dairy productivity can be improved through the molecular selection of genes controlling the interest traits. A genome-based selection approach allows simultaneous selection and improves efficiency compared with traditional or phenotype-based selection [1]. This approach is widely known as marker-assisted selection, which enables the detection of genetic potential down to the deoxyribonucleic acid (DNA) level in the early stages of the life of dairy cows, and its relationship with economically important traits in the dairy industry can also be predicted. The gene involved in dairy cows’ productivity and quality traits, including milk composition, is POU class 1 homeobox 1 (*POU1F1*) [2].

*POU1F1* encodes a member of the POU family of transcription factors that regulate mammalian development. This protein regulates the expression of several genes involved in pituitary gland development and hormone expression in several species of mammals [3, 4]. The protein produced by *POU1F1* can control the expression of genes encoding growth hormone, prolactin, thyrotropin β/thyroid-stimulating hormone β subunit, and gonadotropin-releasing hormone [5]. Previous studies have reported that polymorphisms in *POU1F1* are associated with growth traits in Limousin cattle [6], protein content [7], and fat percentage [8] in milk, and the AB genotype was favorable for increasing fat yield in HF cattle. Different genotypes of *POU1F1* significantly affected total milk production [7], reproductive traits in Holstein cows [9], and milk production traits in Sahiwal cows [10]. Variations in *POU1F1* expression are significantly associated with milk protein and fat yield in Iranian HF cows [11]. To date, research on the association between *POU1F1* and milk quality (physicochemical properties) in Indonesian HF cows has not been reported yet. Therefore, this study evaluated polymorphisms in *POU1F1* in exons 2, 3, and 6 and their associations with the physicochemical properties of milk in high-producing HF cows.

## Materials and Methods

### Ethical approval

The experimental procedures and care of HF cows were approved by the Ethical Clearance Commission of the Faculty of Veterinary Medicine, Universitas Gadjah Mada (Approval no. 057/EC-FKH/Eks./2022).

### Study period and location

This study was conducted from June to November 2023. Blood samples were collected from cows at the PT Ultra Peternakan Bandung Selatan (Pangalengan, West Java, Indonesia). Laboratory work was performed at Universitas Sebelas Maret.

### Dairy population and management

This study involved 149 HF cows from a high-production group reared at PT Ultra Peternakan Bandung Selatan. The inclusion criteria were lactating dairy cows in the 1^st^ and 2^nd^ months after calving. The cows were intensively raised in a closed-house system. Feed was provided in accordance with the daily needs of dairy cows by providing a total mixed ratio. Feed was given once a morning, with both the quantity and quality of the feed adjusted to individual nutritional requirements. Drinking water was provided *ad libitum*. The milking process was routinely performed 3 times a day at 07.00 in the morning, 15.00 in the afternoon, and 23.00 at night.

### Physicochemical properties of milk

To measure the physicochemical properties, milk samples were obtained by collecting 100-mL milk from cows that were milked. The sample from each cow was poured into a collecting bottle, and the identity of each dairy cow was recorded. The physicochemical properties of milk, such as density, freezing point, pH, lactose, solid non-fat, fat, protein, and ash content, were then measured using the Lactoscan SAP tool (Nova Zagora, Bulgaria). The physicochemical properties of the milk were measured at the time of each milking.

### Blood collection and DNA extraction

Blood sampling began by sorting the cows based on their identification numbers by passing the dairy cows through a route equipped with radio-frequency identification. Dairy cows that met the criteria were directed to squeeze chutes for blood collection. Bovine blood (5 mL) was collected from the *coccygeal vein* using a 21-G Vacutainer needle and collected into a vacuum blood collection tube containing the anticoagulant ethylenediaminetetraacetic acid. The samples were then stored in a refrigerator at 4°C until further analysis.

DNA was extracted using a high-salt extraction method developed by Montgomery and Sise [12]. A total of 300 μL bovine whole blood was poured into 1.5 mL and 300 μL red cell lysis buffer solution (pH 7.2) was added and vortexed for 10 min. The samples were centrifuged at 1,000× *g* for 10 min at 10°C. The supernatant was then discarded, and 300 μL red cell lysis buffer solution was added to the pellet left behind, followed by the above steps. This process was repeated 3 times. Subsequently, 300 μL tris buffer saline (TBS) pH 7.4 was added to the microtube containing the pellet, and the mixture was shaken. The mixture was centrifuged at 1,000× *g* for 10 min at 10°C. The supernatant was discarded, and the pellet was processed again using the same procedure.

Moreover, 200 μL TBS was added to the microtube containing the pellet and gently shaken. The samples were centrifuged at 1,000× *g* for 10 min at 10°C, and the supernatant was discarded. The pellet was processed again by adding 200 μL TBS. The sample was gently shaken and centrifuged at 1,000× *g* for 10 min at 10°C. The pellet was then dried, and 2 mL of TE (pH 8.0) was added. To eliminate protein contamination, 200 μL proteinase K solution was added to the mixture, which was incubated at 37°C and 0.91× *g* overnight. After incubation, 200 μL 5M NaCl solution was added. The samples were shaken and centrifuged at 1,000× *g* for 10 min at 10°C. In addition, 400 μL supernatant was transferred to a new 1.5 mL microtube and 800 μL cold absolute alcohol was added. The mixture was centrifuged at 10,000× *g* for 10 min at 10°C, and the supernatant was discarded. To the pellet, 800 μL 70% alcohol was added and centrifuged at 10,000× *g* for 10 min at 10°C. The supernatant was discarded, and the pellet was dried at room temperature (33°C). In addition, the pellet was dissolved with 100 μL nuclease- and RNAse-free water. Finally, the extracted DNA was analyzed by polymerase chain reaction (PCR).

### Amplification of *POU1F1*

Three polymorphisms targeting exons 2, 3, and 6 of *POU1F1* (c.195G>A, c.300G>T, and c.828G>A, respectively, were amplified using a thermal cycler machine (MiniAmp Plus Thermal Cycler, Singapore). PCR was conducted in 25 μL total reaction volume containing 12.5 μL PCR Master Mix (My Taq HS Red Mix 2× Bioline, London, UK), 9.5 μL nuclease-free water (1^st^ BASE, Singapore), 1 μL DNA template, and 1 μL of each primer (Table-1). DNA amplification was initiated by pre-denaturation at 94°C for 3 min followed by 35 cycles of denaturation (94°C, 30 s), annealing for 30 s (Table-1), and extension (72°C, 30 s). PCR was performed with a final extension (72°C, 10 min). The PCR products were visualized using agarose gel electrophoresis (Advance, Tokyo, Japan) at 110 V for 30 min. A 2% agarose gel was then stained with ethidium bromide (Promega, WI, USA). A 100-bp marker ladder (Geneaid, Taiwan) was used as the standard DNA band size. Images of the agarose gels were captured using a gel documentation system (Glite UV, Pacific Image, Taiwan) under ultraviolet (UV) light.

**Table-1:** Primer pairs targeting exons 2, 3, and 6 of *POU1F1*.

Primer	Target	Product size (bp)	Ta (°C)	Restriction enzyme
F: 5’- cttaccagtcccgtctatt-3’	Exon 2	165	51	*Taq*I
R: 5’- ttcttacctgccatcacg-3’				
F: 5’- ccttttagaactgagactggctg-3’	Exon 3	356	58	*Eco*O109I
R: 5’- cccacagctgttaacaagca-3’				
F: 5’- aaaccatcatctcccttctt-3’	Exon 6	451	51	*Hinf*I
R: 5’- aatgtacaatgtgccttctgag-3’				

F=Forward primer, R=Reverse primer, Ta=Annealing temperature

### Genotyping of *POU1F1*

Polymorphisms in *POU1F1*, c.195G>A, c.300G>T, and c.828G>A were genotyped using PCR-restriction fragment length polymorphism. Each PCR product was respectively digested with Fast Digest *Taq*I for c.195G>A single-nucleotide polymorphisms (SNP) at 65°C for 2 h, Fast Digest *Eco*O109I for c.300G>T SNP at 37°C for 2 h, and Fast Digest *Hinf*I for c.828G>A SNP at 37°C for 2 h (Thermo Fisher Scientific Inc., Vilnius, Lithuania). The reaction mixture contained 10 μL PCR product, 18 μL nuclease-free water, 2 μL 10× buffer tango, and 0.1 μL of each restriction enzyme. The digested PCR products were analyzed by 25 agarose gel electrophoresis at 100 V for 35 min. The agarose gel was stained with Florosafe DNA (1^st^ BASE, Malaysia) and visualized under UV light using a gel documentation system. A 100-bp DNA ladder was used as the standard DNA band size.

### Hardy-Weinberg equilibrium (HWE) analysis

Both genotype and allele frequencies were calculated using the Hardy–Weinberg equation. The Chi-square test was used to evaluate the observed and expected genotype and allele frequencies. The Hardy–Weinberg equation is as follows:

p + q = 1

p^2^ + 2 pq + q^2^

Where p is the first allele frequency and q is the other allele frequency. In addition, Chi-square test was conducted using the following formula:


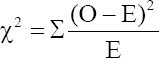


χ^2^ is Chi-square, O is the observed genotype frequency, and E is the expected genotype frequency [13]. Both genotype and allele frequencies meet the Hardy–Weinberg principle if the χ^2^ value at 5% alpha is <3.84.

### Associations of *POU1F1* polymorphisms with the physicochemical properties of milk

A normality test was performed to determine whether the data were distributed normally. A mixed model was used to evaluate the effect of *POU1F1* polymorphisms on the physicochemical properties of milk using Minitab 19.0 (www.minitab.com). The model included the fixed effects of *POU1F1* genotypes and random effects of sire and lactation period as covariates. Fisher LSD method was applied to determine pairwise comparison among genotypes.

## Results

### Profile of the physicochemical properties of milk

Table-2 presents the descriptive statistics of the physicochemical properties of Indonesian HF cow milk. The physicochemical properties of milk were normally distributed, except for milk fat content, which was excluded from the association analyses.

**Table-2:** Profile of milk physicochemical properties of Indonesian Holstein Friesian cows.

Trait	n	Mean	SEM	Minimum	Maximum
Density (kg/m^3^)	149	1030.11	0.365	1012.20	1035.40
Lactose (%)	149	4.66	0.032	3.11	5.26
SNF (%)	149	8.50	0.057	5.72	9.56
Protein (%)	149	3.11	0.022	2.03	3.51
Freezing point (°C)	149	−0.53	0.003	−0.598	−0.381
Ash (%)	149	0.69	0.005	0.46	0.78
pH	149	6.54	0.007	6.35	6.78

n=Number of samples, SEM=Standard error of the mean, SNF=Solid not fat

### Amplification and genotyping of *POU1F1* polymorphisms

**Figure-1:**
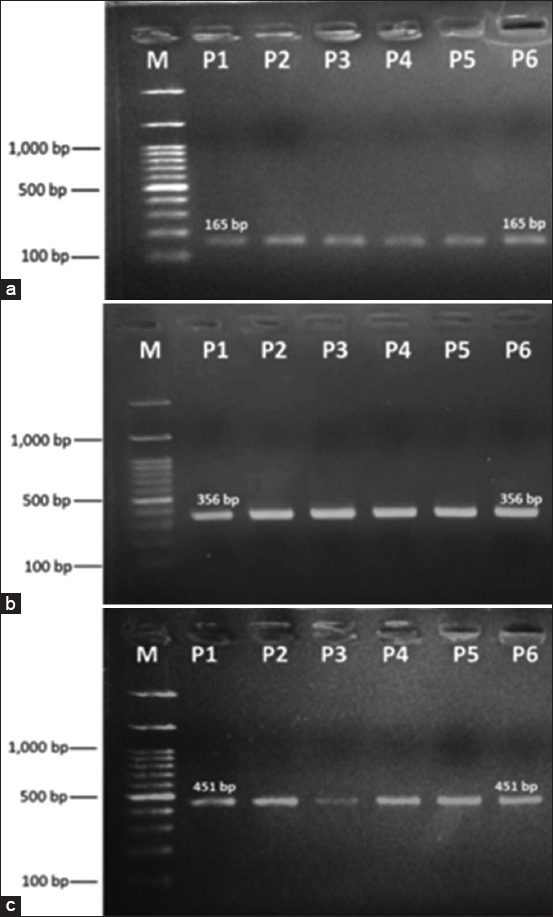
PCR products targeting exonic mutations in POU1F1. (a) PCR product to identify c.195G>A SNP in exon 2; (b) PCR product to identify c.300G>T SNP in exon 3; (c) PCR product to identify c.828G>A SNP in exon 6. M is a 100-bp marker ladder, P1-P6 are Holstein Friesian cow samples. PCR=Polymerase chain reaction, SNP=Single nucleotide polymorphisms.

**Figure-2:**
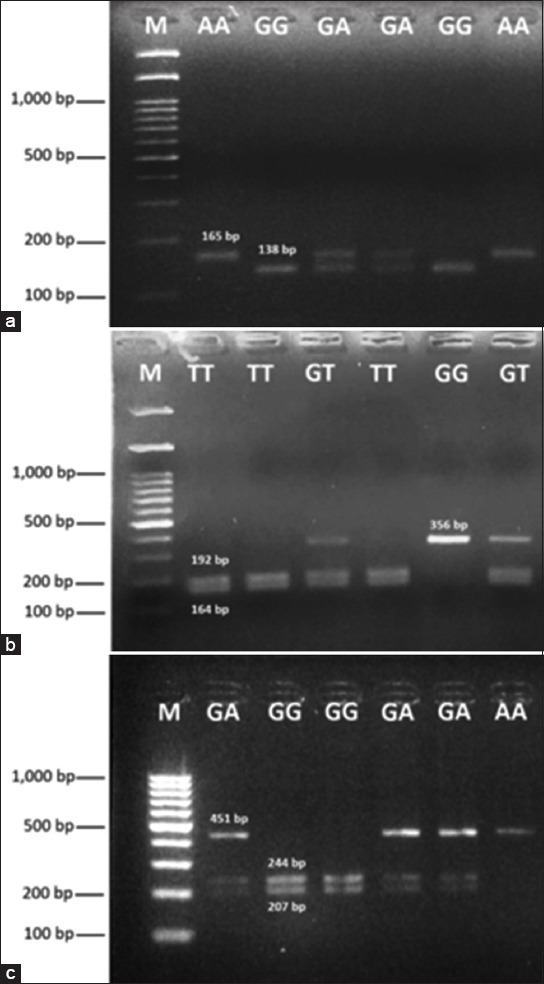
Polymerase chain reaction-restriction fragment length polymorphism products of *POU1F1* digested by *Taq*I, *Eco*O109I, and *Hinf*I. (a) Genotypes of c.195G>A SNP in exon 2; (b) Genotypes of c.300G>T SNP in exon 3; (c) Genotypes of c.828G>A SNP in exon 6. M is a 100-bp marker ladder. SNP=Single nucleotide polymorphisms.

Three target polymorphisms of *POU1F1* were amplified to yield 165, 356, and 451 bp PCR products (Figure-1). The PCR product size for detecting the c.195G>A SNP in exon 2 of *POU1F1* is 165 bp (Figure-1a), c.300G>T SNP in exon 3 is 356 bp (Figure-1b), and c.828G>A SNP in exon 6 is 451 bp. The product sizes were in accordance with estimated PCR product sizes when the primer pair was designed to identify the c.300G>T SNP and the references for the c.195G>A and c.828G>A SNPs. In addition, all genotypes of the three SNPs were observed (Figure-2). Genotypes of the c.195G>A SNP were GG (138 and 27 bp), GA (165, 138, and 27 bp), and AA (165 bp) (Figure-2a); Three genotypes of the c.300G>T SNP were GG (356 bp), GT (356, 192, and 164 bp), and TT (192 and 164 bp) (Figure-2b); and three genotypes of the c.828G>A SNP were GG (244 and 207 bp), GA (451, 244, and 207 bp), and AA (451 bp) (Figure-2c).

### Genotype and allele frequencies of *POU1F1* polymorphisms

The genotypes and allele frequencies of the three SNPs in Indonesian HF cows are shown in Table-3. Only the c.195G>A SNP followed the Hardy–Weinberg principle. Contrarily, the genotype and allele frequencies of the c.300G>T and c.828G>SNPs were not in HWE indicated by significance χ^2^ value (p < 0.05).

**Table-3:** Allele and genotype frequencies of *POU1F1* SNPs.

SNP	c.195G>A		c.300G>T		c.828G>A	
Genotype frequency	GG	0.531	GG	0.161	GG	0.497
	GA	0.415	GT	0.651	GA	0.342
	AA	0.054	TT	0.188	AA	0.161
Allele frequency	G	0.738	G	0.487	G	0.668
	A	0.262	T	0.513	A	0.332
*χ* ^2^		0.79		13.675		7.784
p-value		0.374		<0.05		<0.05

*χ*^2^=Chi-square, SNP=Single nucleotide polymorphism

### Association between *POU1F1* polymorphisms and milk physicochemical properties

The statistical analysis showed that two *POU1F1* polymorphisms were significantly associated with the pH of milk (Table-4). Three genotypes of the c.195G>A SNP were detected, and the milk of cows with the GG genotype had a higher pH. Three genotypes were identified for the c.828G>A SNP. The lowest milk pH was observed in Indonesian HF cows with the GG genotype. In contrast, the c.300G>T SNP was not significantly associated with any physicochemical properties of milk. The *POU1F1* polymorphisms evaluated in this study did not significantly affect the other physicochemical properties of milk.

**Table-4:** Association between *POU1F1* SNPs and the physicochemical properties of milk.

SNP	Genotype	Density (kg/m^3^)	Lactose (%)	SNF (%)	Protein (%)	FP (°C)	Ash (%)	pH
c.195G>A	GG (78)	1029.9	4.65	8.47	3.10	−0.530	0.687	6.51^b^
	GA (61)	1030.5	4.70	8.55	3.13	−0.536	0.694	6.56^a^
	AA (8)	1030.5	4.70	8.55	3.13	−0.536	0.694	6.61^a^
p-value		0.914	0.915	0.934	0.916	0.907	0.940	**0.001**
	GG (24)	1028.8	4.55	8.28	3.03	−0.521	0.675	6.55
c.300G>T	GT (97)	1030.2	4.68	8.52	3.12	−0.534	0.691	6.53
	TT (28)	1030.8	4.70	8.58	3.14	−0.535	0.694	6.55
p-value		0.187	0.221	0.211	0.215	0.262	0.339	0.337
c.828G>A	GG (74)	1030.0	4.65	8.48	3.10	−0.531	0.688	6.52^b^
	GA (51)	1030.8	4.71	8.58	3.14	−0.537	0.696	6.56^a^
	AA (24)	1029.4	4.60	8.37	3.06	−0.526	0.679	6.56^a^
p-value		0.159	0.176	0.177	0.166	0.186	0.880	**0.029**

SNP=Single nucleotide polymorphism, SNF=Solid non-fat, FP=Freezing point ^a,b^different superscript in the same column indicates significantly different among genotypes. bold p-values represent significant effect of genotype.

## Discussion

Three *POU1F1* polymorphisms, c.195G>A, c.300G>T, and c.828G>A, were evaluated for their associations with the physicochemical properties of milk in high-producing HF dairy cow populations. Polymorphism was found to occur, and all three genotypes of each SNP were identified in this study. The G allele frequency of the c.195G>A SNP in exon 2 was higher than that of the A allele in this population. Previous studies have reported that the G allele frequency was higher than that of the A allele in various cattle breeds in China [14], Podolica cattle in Italy [15], HF cattle in Vietnam [16], and HF cattle in Iran [17]. Considering the c.828G>A SNP in exon 6, the G allele frequency was higher than the A allele frequency in this population, which is similar to the findings of Renaville *et al*. [18] (in Italian HF bulls) and Thuy *et al*. [16] (in HF dairy cows in Vietnam). In addition, the frequency of the T allele of the c.300G>T SNP in exon 3 was higher than that of the G allele. This is the first report to identify c.300G>T using the *Eco*O109I restriction enzyme *in vitro*, whereas an *in silico* analysis of this SNP was previously reported by Nissa *et al*. [19]. The genotype and allele frequencies of these SNPs were calculated to evaluate whether they followed the HWE principle. The c.300G>T and c.828G>SNPs did not follow the HWE principle. Contrarily, the c.195G>A SNP followed the HWE principle indicated by the non-significance of the Chi-square (*χ^2^*) test (p = 3.374). Saadat [20] explained that a population follows the HWE principle when there is no natural selection, no migration, no mutation, and the locus is located on a non-sex chromosome (autosome).

The physicochemical properties of milk in the HF cattle population observed in this study were not significantly affected by exonic mutations in *POU1F1*, except for the pH value. This finding differs from a previous study by Renaville *et al*. [18], who reported that *Hinf*I polymorphism in exon 6 of *POU1F1* significantly affected milk protein content in Italian HF cattle. Aytekin and Boztepe [21] investigated the association between *POU1F1* *Hinf*I polymorphism and milk pH in Brown Swiss cattle. Their results showed that this gene cannot be used as a candidate gene for selecting dairy traits. Therefore, our study provides an update on the relationship between *POU1F1* polymorphisms and the pH of fresh milk. HF cattle with the A alleles *Taq*I (c.195G>A) and *Hinf*I (c.828G>A) produce milk with higher pH values.

pH, the degree of acidity, is defined as the logarithm of hydrogen ion activity and indicates the concentration of hydrogen ions. Normally, the pH of milk ranges from 6.5 to 6.7, with 6.6 being the most common value [3]. A decrease in pH from the normal range may cause changes in the composition of milk components, owing to the breakdown of colloidal calcium phosphate and reduced bonds between cations and proteins [22]. Different pH values can affect the properties of milk products. Ozcan *et al*. [23] found that different pH values in fresh milk significantly affected the physical properties of yogurt. The optimal pH of fresh milk for yogurt-making is 6.7, which results in yogurt with a viscous and smooth texture because it contains a balance between bound and dissolved nutrients [23, 24]. Based on the abovementioned data, dairy cows with the A alleles c.195G>A and c.828G>A SNPs produced milk with higher pH values (6.56–6.61); therefore, their milk is recommended for use as a raw material for yogurt production. In addition, milk pH affects the texture, dry weight, and curd yield of cheddar cheese [25]. Therefore, this factor is important in cheese manufacturing [26]. Milk with an initial pH of 6.3–6.5 can improve cheese dry matter yield without concurrent changes in texture. Moreover, the low pH of milk affects the texture of cream cheese. A higher pH produces cream cheese with a soft texture, whereas a lower pH produces a grainy texture [27]. Therefore, milk from dairy cows with the GG genotype can produce cheddar and cream cheeses with a soft texture. Different properties, such as casein hydration, colloidal calcium, and electrostatic charges of proteins, are also affected by milk pH [28]. In addition, changes in milk pH affect protein charge, conformation, and stability during denaturation [27, 29].

## Conclusion

The results of this study imply that c.195G>A and c.828G>A SNPs of *POU1F1* in the milk of high-producing HF dairy cows are associated with the pH value of their milk. Cows with the GG genotype produced milk with lower pH values than those with the AA genotype. *POU1F1* is a potential candidate marker for dairy cow selection, particularly for milk pH. These results may benefit breeding programs and product development in the dairy and milk processing industries.

## Authors’ Contributions

MC: Conceptualization, supervision, investigation, and drafted and revised the manuscript. AIH, DSS, and NAN: Investigation, data curation and drafted the manuscript. AP: Supervision and edited the manuscript. SF and RNF: Methodology and data curation. SDV: Data curation. PS: Validation and drafted and edited the manuscript. All authors have read and approved the final manuscript.
